# Understanding How to Make Type 2 Diabetes Prevention Programs More Inclusive: An Equity-Oriented Qualitative Case Study of Small Steps for Big Changes

**DOI:** 10.3390/healthcare14203432

**Published:** 2026-10-14

**Authors:** Sarah Der, Kaela Cranston, Mary Jung

**Affiliations:** School of Health and Exercise Sciences, Faculty of Health and Social Development, University of British Columbia, Okanagan Campus, Kelowna, BC V1V 1V7, Canada; seder@ualberta.ca (S.D.); kaela24@mail.ubc.ca (K.C.)

**Keywords:** type 2 diabetes, prediabetes, diabetes prevention program, health equity, social determinants of health, qualitative case study, cultural tailoring, young adults, South Asian, motivational interviewing

## Abstract

**Highlights:**

**What are the main findings?**

- A young adult South Asian, male-identifying participant experienced prediabetes prevention as a problem of access, not only individual motivation: culturally irrelevant nutrition resources, student food environments, cost, long waits for dietetic support, isolation and mental health strain shaped engagement.
- The one-on-one motivational interviewing-informed coach was experienced as supportive, non-judgmental and motivating. Still, the standard program did not fully address cultural food practices, young adult routines, peer connection or coping with prediabetes-related distress.

**What are the implications of the main findings?**

- Equity-oriented DPP improvement should move beyond recruitment diversity and add modular cultural nutrition resources, peer/community options, mental health referral pathways and routine review of age-, culture-, and cost-related barriers.
- The case is not statistically generalizable; its contribution is analytic and implementation-focused, identifying mechanisms through which standardized prevention programming can unintentionally reproduce exclusion.

**Abstract:**

**Background/Objectives:** Diabetes prevention programs (DPPs) reduce type 2 diabetes (T2D) risk, yet their reach often remains misaligned with the populations most affected by social and structural diabetes risk. This study examined inclusivity barriers within Small Steps for Big Changes (SSBC), a Canadian DPP developed at the University of British Columbia, through an in-depth qualitative case study of a participant whose age, ethnicity and gender identity differed from the program’s typical participant profile. **Methods:** The participant was purposively selected for the present case analysis from individuals enrolled in SSBC and completed the standard 4-week intervention, consisting of one-on-one motivational interviewing-informed counseling, supervised exercise, fitness-tracker- and mobile-app-supported self-monitoring, and planned long-term follow-up. Data were collected from two semi-structured Zoom interviews, six session-day journal entries and two optional non-session journal entries between July and September 2022. Data were analyzed in NVivo using interpretive description. **Results:** The participant was a young South Asian adult who self-identified as male. Five themes were identified: lack of access, limitations and barriers, lack of support, mental health implications, and SSBC-related takeaways. Barriers included culturally irrelevant nutrition resources, the university food environment, cost constraints, a one-year wait for dietetic care, isolation from peers managing prediabetes, and prediabetes-related guilt and stress. Supportive, non-judgmental coaching strengthened accountability and motivation but did not fully address culture-, age-, student-life-, and mental-health-related needs. **Conclusions:** This case identifies practical equity targets for DPP redesign, including culturally adaptable dietary resources, peer support, inclusive coaching training and mental health-responsive prevention care.

## 1. Introduction

Type 2 diabetes (T2D) remains a major and increasing global health burden. The International Diabetes Federation reported that 589 million adults aged 20–79 years were living with diabetes in 2025, equivalent to approximately one in nine adults, and projected this number to rise to 853 million by 2050; diabetes was also associated with at least USD 1 trillion in health expenditure and 3.4 million deaths in 2024 [1]. Earlier global epidemiological syntheses similarly anticipated a sustained increase in diabetes prevalence and health-system burden [2]. In Canada, approximately 3.9 million people aged one year and older live with diagnosed diabetes, representing 9.7% of the population, and more than 6% of adults live with prediabetes [3]. The age-standardized prevalence of diagnosed diabetes in Canada was 9.40% in 2023–2024 [4].

Prediabetes is a high-risk metabolic state in which glycemic measures are above normal but below diabetes diagnostic thresholds. Diagnostic thresholds vary across guidance: Diabetes Canada defines prediabetes using a higher-risk A1C range of 6.0% to 6.4%, whereas the American Diabetes Association includes A1C 5.7% to 6.4% [5,6]. This distinction matters for Canadian prevention studies because program eligibility may use broader American Diabetes Association risk criteria while clinical interpretation in Canada often relies on Diabetes Canada thresholds. Prediabetes is associated with insulin resistance, beta-cell dysfunction and a 5–10% annual risk of progression to diabetes, although progression varies by glycemic category, age, adiposity, family history and social context [7,8]. Landmark prevention trials showed that structured lifestyle interventions can reduce diabetes incidence, including a 58% relative reduction in the original U.S. Diabetes Prevention Program trial, with durable benefits also reported in the Da Qing and Finnish Diabetes Prevention studies [9,10,11].

The effectiveness of lifestyle prevention is not the same as equitable reach. Diabetes risk and diabetes outcomes are patterned by social determinants of health, including income, education, food access, neighborhood environment, racism, health-care access and social support [12,13,14]. In Canada, First Nations and Metis people and people of African, East Asian and South Asian ethnic backgrounds experience higher rates of T2D compared with the general population [13]. Socioeconomic gradients also affect glycemic control and diabetes mortality in Canadian populations [15,16], and the Public Health Agency of Canada has identified persistent health inequalities related to income, education, Indigenous identity, ethnicity and other structural determinants [17].

Despite this burden, many DPPs have historically reached relatively advantaged and demographically narrow groups. U.S. National DPP data show that participants have been disproportionately female, middle-aged and non-Hispanic White, with more than 60% identified as non-Hispanic White in early implementation analyses [18]. Retention also varies by age and race/ethnicity, with lower retention reported among younger adults and among Black, Hispanic and other racial/ethnic groups compared with non-Hispanic White participants [19]. Reviews of real-world DPP implementation have therefore argued that improving reach among underserved populations requires more than translating materials; it requires attention to social needs, trust, cultural relevance, cost, access and delivery context [20]. Recent evidence specific to South Asian populations also supports culturally bespoke lifestyle approaches, while emphasizing that tailored interventions remain heterogeneous and not always sufficient to eliminate response gaps [21].

Small Steps for Big Changes (SSBC) is an evidence-based, community-oriented diabetes prevention program developed at the University of British Columbia to help individuals at risk of T2D adopt sustainable diet and exercise behaviors. Prior SSBC evaluation demonstrated promising effectiveness among enrolled participants but also documented a narrow participant profile: in a community-site evaluation, 213 adults with prediabetes enrolled, 87.4% were Caucasian, 69.7% were female, and program completion was 95% [22]. Intervention reporting has identified 43 behavior change techniques and 20 motivational interviewing techniques within SSBC protocols, supporting replicability and scale-up [23]. More recent equity work has described SSBC efforts to improve inclusive access through stakeholder advisory input and the ConNECT Framework [24].

The present study extends this literature by shifting from program-level reach and adaptation to a longitudinal participant-level case analysis. The novelty is deliberately bounded: this is not a prevalence study and does not claim to be representative. Its value lies in identifying concrete, implementation-relevant mechanisms through which a standardized DPP can fit some participants while remaining only partially responsive to others. We used an in-depth qualitative case study to examine the experience of a young South Asian, male-identifying SSBC participant, focusing on how culture, age, student life, financial constraints, social support and mental health interacted with program engagement. Gender identity was included as a prompt-directed area of inquiry but was not identified in the participant data as a specific barrier to engagement. The objective was to generate actionable refinements that could strengthen equity, reach and inclusivity in SSBC and comparable DPPs.

## 2. Materials and Methods

### 2.1. Study Design and Reporting Orientation

This study used a qualitative case study design within a process and outcome evaluation of SSBC. Reporting was guided by principles from the Standards for Reporting Qualitative Research and, where applicable, the Consolidated Criteria for Reporting Qualitative Research [25,26]. The analytic approach was interpretive description, selected because it is designed to generate clinically and practically relevant understanding from subjective health and illness experiences rather than only produce abstract theory [27].

### 2.2. Setting and Intervention

SSBC is a Canada-wide DPP developed at the University of British Columbia. Key partners included SSBC staff, including coaches and coordinators, and UBC researchers. The standard intervention was delivered without modification between July and September 2022, with concurrent data collection. All SSBC participants receive the same standard program via an open waitlist.

The intervention is designed to empower individuals to make sustainable diet and exercise changes to reduce their risk of developing T2D. It includes a baseline appointment, followed by a 4-week intensive phase consisting of motivational interviewing-based counseling and supervised exercise sessions, as well as a 12-month follow-up phase designed to support longer-term behavior change. The participant used a fitness tracker and mobile self-monitoring app during the intervention to monitor progress and support health behavior change. The intervention emphasizes autonomy, self-management, and accountability. Although the standard SSBC program includes a 12-month follow-up phase, the present case study did not evaluate outcomes during this period. The qualitative data analyzed in this study were limited to the 4-week intensive intervention and the immediate post-intervention assessment.

For this equity-oriented analysis, we examined how the standard model assumes that participants can apply broadly framed diet and exercise recommendations, that one-on-one motivational coaching can enhance adherence, and that behavior change can be sustained with follow-up support. These assumptions are clinically plausible, but they may operate unevenly when participants face culturally specific dietary patterns, limited food choice, financial constraints, student schedules, isolation or mental health stressors.

### 2.3. Rationale for Case Selection

Previous evaluations of SSBC identified a relatively homogeneous participant profile, predominantly female, White, and of middle socioeconomic status. Within the broader SSBC program evaluation, participant experiences were collected to better understand the relevance and inclusivity of the standard intervention across participants with different backgrounds. The present case was selected retrospectively for in-depth analysis because the participant’s characteristics differed from those historically represented in SSBC, including younger age, male gender identity, and non-White racial or ethnic background. The single-case design was used to provide an in-depth, longitudinal account of this participant’s experience rather than to make comparisons across participants or demographic groups.

### 2.4. Participant Recruitment and Eligibility

The participant had been enrolled in SSBC through the program’s existing recruitment and eligibility process. For the present case analysis, the participant was purposively selected because they were 18–35 years of age, self-identified as male and non-White, and were able to speak, read, and write English. SSBC program eligibility was established by either (1) scoring at risk on the American Diabetes Association questionnaire or (2) having an HbA1c level between 5.7% and 6.4%, confirmed by a medical laboratory. This HbA1c range reflects the American Diabetes Association prediabetes threshold and is broader than the Diabetes Canada HbA1c prediabetes criterion of 6.0–6.4% [5,6]. For the participant included in this case study, SSBC eligibility was established based on a laboratory-confirmed HbA1c within the 5.7–6.4% range, obtained through their family physician, rather than through the ADA risk questionnaire.

### 2.5. Procedures and Measures

The participant provided written informed consent and completed a demographic survey to confirm eligibility. Data were collected through two semi-structured interviews and a series of journal entries. The first interview, conducted over Zoom before the participant started SSBC, explored experiences with diet, exercise, prediabetes and goals for the program. The second interview, conducted after the intervention, gathered reflections on the participant’s overall experience and feedback on SSBC. The interviews lasted approximately 45 and 30 min, respectively.

Throughout the program, the participant completed one journal entry per session using Qualtrics, with the option to submit additional entries on non-session days. Session-specific prompts focused on the week’s topic, while non-session prompts encouraged reflection on how participation in SSBC influenced daily diet and exercise behaviors. Journal prompts addressed the first coaching session, sugar, exercise, carbohydrates, additional exercise content and success after SSBC, including prompts about cultural relevance, age relevance, gender identity relevance and missing content (Supplementary File S1). Interview and journal prompts were drafted by the research team based on the study’s equity-oriented objectives and the SSBC intervention structure, to elicit participants’ experiences of the SSBC intervention, including perceived relevance related to culture, age, and gender. Prompts were reviewed internally by the research team for clarity and relevance before use; no external expert panel or formal pretesting was conducted. The guides were developed as qualitative data-collection tools for this case study rather than standardized psychometric instruments, so formal psychometric validation was not undertaken. The interview and journal guides included a priori prompts concerning cultural relevance, age relevance, gender identity relevance, and perceived gaps in program content; these domains were therefore treated as prompt-directed areas of inquiry rather than independently emergent findings. Analysis remained open to additional patterns and issues raised by the participant beyond these predefined domains.

### 2.6. Analysis

**Figure 1:**
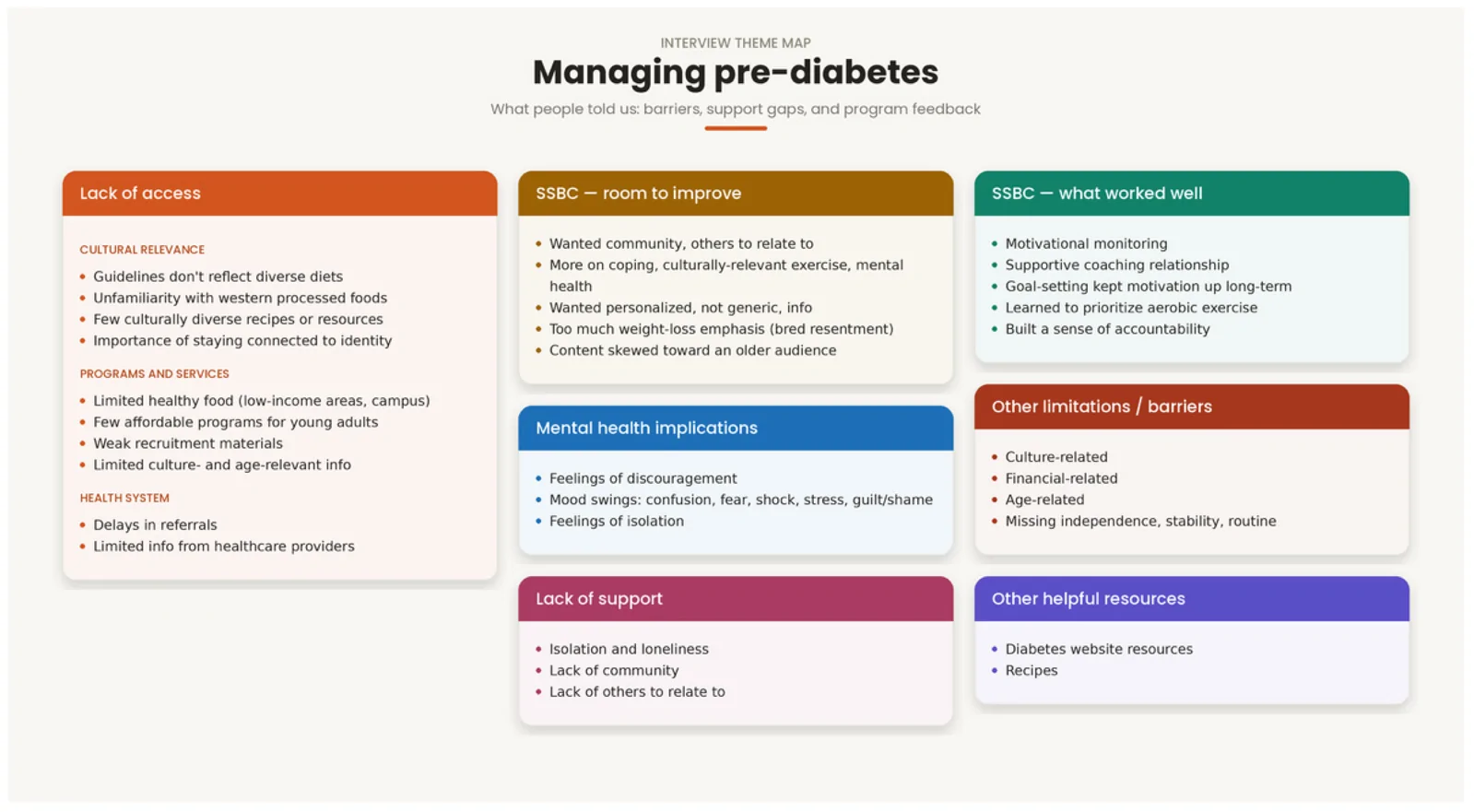
Thematic summary of participant-reported factors related to managing prediabetes, grouped into seven categories: lack of access, other limitations/barriers, lack of support, mental health implications, other helpful resources, and SSBC-related positive takeaways and areas for improvement.

Semi-structured interviews were audio-recorded on Zoom and transcribed by the first author. Interview transcripts and journal responses were imported into NVivo 12 for analysis using interpretive description. Coding used a combined deductive and inductive approach: the interview and journal guides included predefined areas of inquiry related to culture, age, gender identity, and perceived gaps in program content, while coding remained open to additional patterns arising from the participant’s responses. SD and KC independently reviewed the qualitative data and assigned codes to relevant segments of the interview transcripts and journal entries. The two researchers then compared their coding and discussed differences in interpretation until consensus was reached. Related codes were grouped to develop candidate themes representing recurring barriers, facilitators, and contextual influences on the participant’s experience. Candidate themes were subsequently discussed and refined by the researchers, with consideration of patterns across both interview and journal data, resulting in the five final themes presented in the Results. Interview and journal data were considered together to examine the participant’s experiences before, during, and immediately following the 4-week intervention. A concept map was developed to illustrate relationships among the final themes (Figure 1).

### 2.7. Reflexivity, Trustworthiness and Ethics

Trustworthiness was supported through multiple qualitative data sources collected across the study period, including pre-intervention and post-intervention interviews, six session-day journal entries, and two optional non-session journal entries. SD conducted the qualitative interviews but had no role in delivering the SSBC intervention. KC had direct contact with the participant during the initial program recruitment and consent process but did not conduct the qualitative interviews or deliver the intervention. MJ did not have direct interaction with the participant during recruitment, data collection, or intervention delivery. KC held an administrative role within the SSBC team, and MJ was the principal investigator of the research laboratory and founder of SSBC. These roles provided the research team with contextual knowledge of the intervention while also creating the potential for their positions within the program to influence interpretation.

To address this potential influence, SD and KC independently reviewed and coded the qualitative data before comparing their interpretations and resolving differences through discussion and consensus. Themes were grounded in the participant’s interview and journal data, and interpretations considered both positive experiences and perceived limitations of SSBC. The use of multiple data sources across time further supported comparison of the participant’s experiences before, during, and immediately following the intervention. Given the single-case design, findings are presented as an in-depth, hypothesis-generating account rather than as representative of broader populations.

Participant engagement in the SSBC program, including the interviews and journal entries analyzed here, was conducted under the program’s standing institutional ethics approval (approved 11 April 2022), which permitted participants to complete the intervention and take part in associated program-evaluation activities. At the time of data collection (July–September 2022), this was conceived as an internal program evaluation and, per UBC Behavioural Research Ethics Board (BREB) criteria, was not considered to require separate research ethics review. As the value of these data for broader dissemination became apparent, the team subsequently sought formal BREB approval (H22-02212, approved 29 November 2022) to analyze and publish the data; this approval was obtained retrospectively, with the participant’s consent, prior to any formal analysis or publication. The participant’s written informed consent covered both interview participation and use of de-identified data for publication; the team subsequently reviewed the demographic detail reported here, judged it necessary to convey the case’s equity relevance, and removed identifiers not essential to interpretation (e.g., specific university, program site, exact interview dates). The qualitative data are available on reasonable request from the corresponding author but are not publicly available due to their sensitive and potentially identifiable nature (Table 1).

## 3. Results

The participant completed all six session-day journals and two additional non-session entries. Five themes were identified: lack of access, limitations and barriers, lack of support, mental health implications, and SSBC-related takeaways. The themes were connected rather than isolated: food access shaped guilt and stress; culturally irrelevant resources limited family-level implementation; student life intensified financial and schedule barriers; and the absence of age-similar peers amplified isolation. Figure 1 summarizes the concept map developed from the qualitative analysis.

### 3.1. Lack of Access

Two related but distinct sets of barriers emerged: lack of access refers to external, systemic gaps in available resources and services, while limitations and barriers (Section 3.2) refer to life-stage and resource-related constraints that shaped how the participant could act within those systems.

Culturally appropriate resources and information. The participant reported that non-Western foods were rarely represented in Western DPP materials. They described “the diabetes website, and the recipes on it [as] very much not catered towards an ethnic diet, it’s very basic,” and added that recipes used “cuisine and flavouring that I don’t think my parents would be eating.” They also identified “a knowledge gap, …, [my family] doesn’t know how to read nutritional values,” highlighting that cultural relevance and nutrition literacy were linked barriers rather than separate problems.

Healthy food as a university student. Living on campus created additional constraints. The participant described cafeteria food as “purely white flour, white paste, red meat, …, [and] a lot of high sugar foods.” They also noted that social events “always [have] free pizza, …, free sugar foods,” which made it “really difficult for me to like, fight those cravings.” These experiences produced not only dietary difficulty but also “overwhelming guilt” and social exclusion, suggesting that food environments can transform prevention advice into a daily psychological burden.

Healthcare providers and health information. The participant waited approximately one year to see a dietician and described having to “figure out stuff by myself” and “pioneer new ways, …, as a student.” Free online resources were described as “not considerate of non-Western cultures.” This theme therefore included both formal health-system delay and the inadequacy of available public-facing resources.

### 3.2. Limitations and Barriers

Age-related barriers. The participant contrasted student life with older adulthood, explaining that “older adults have a lot more routine,…, versus like, living as a student,…, and not having that routine or dependability.” They described eating during young adulthood as something that often had to fit around classes and an unpredictable student schedule, rather than a structured routine. The participant also observed that DPP recruitment materials often targeted “age 40 and up” and used images of older individuals centered around weight, which signaled that the program was not designed with young adults in mind.

Financial barriers. The participant reported, “I couldn’t eat anywhere outside of [the student dining hall] without paying even more of my own money.” Cost therefore constrained the feasibility of applying healthy eating advice, especially when the available low-cost or pre-paid food environment did not align with diabetes prevention recommendations.

### 3.3. Lack of Support

The participant experienced isolation, exclusion and stress related to limited support. They reported not knowing “other young people managing prediabetes.” This lack of peer visibility made prediabetes feel socially atypical for their age group and reduced opportunities for shared problem-solving.

### 3.4. Mental Health Implications

The participant described major emotional shifts after learning of their prediabetes status, including confusion, fear, shock, stress, guilt, shame and isolation. They reported “a significant change in mood right after you switch completely off from sugar” and discussed “finding other sources of joy [than food]” while managing energy and self-control. These accounts show that prevention behavior change was experienced as emotional labor, not only information uptake.

### 3.5. SSBC-Related Takeaways

Positive takeaways. The participant highlighted their coach as “very supportive and non-judgemental.” This coaching relationship fostered a sense of belonging and motivation. Goal setting and regular sessions provided an “accountability factor” and “external motivation.” The participant also reported learning about the value of aerobic exercise for blood glucose control.

Areas for improvement. The participant felt the program “did not target a specific demographic” and was “targeted at people who had never really taken up exercise before.” They recommended adding community connections, non-Western dietary education, culturally relevant exercise options, coping strategies and mental health resources.

### 3.6. Changes Across the Intervention Period

Across the study period, the participant’s experiences demonstrated both continuity and change. Before beginning SSBC, the participant described difficulties accessing culturally relevant nutrition information and dietetic support, as well as challenges related to managing prediabetes as a young adult. During the intervention, regular sessions with the SSBC coach provided a supportive and non-judgmental source of accountability and motivation. However, barriers related to food access, cost, cultural relevance, and student life continued during program participation. In the immediate post-intervention interview, the participant continued to identify the coach’s support and increased knowledge of exercise as positive aspects of SSBC, while also identifying persistent unmet needs related to culturally relevant resources, peer connection, and coping support. Overall, the participant’s account suggests that SSBC provided valued interpersonal and behavioral support during the intervention, while several contextual barriers remained unresolved at the immediate post-intervention assessment (Table 2).

## 4. Discussion

### 4.1. Principal Findings

This case study shows that the participant’s experience of prediabetes prevention was shaped as much by access and support as by personal motivation. Before entering SSBC, the participant had already experienced approximately one year of limited dietetic access, inadequate culturally relevant resources, social isolation and self-reported emotional distress, including stress, guilt and shame. During SSBC, the coach provided meaningful accountability, motivation and non-judgmental support, but the standard intervention did not fully resolve cultural, age-related, financial, student-life and mental health barriers. The most important finding is not simply that the participant wanted “more tailoring.” The sharper interpretation is that standardized DPP delivery can place the burden of adaptation on participants who are already navigating structural constraints.

The case therefore reframes inclusivity as an implementation property rather than an optional add-on. A DPP can be evidence-based and still be unevenly accessible if its food examples, recruitment imagery, scheduling assumptions, peer environment, and resource pathways implicitly fit older, White, middle-socioeconomic-status participants better than younger or racialized participants. This interpretation is consistent with the participant’s observation that the program “did not target a specific demographic” and appeared “targeted at people who had never really taken up exercise before,” while also lacking resources for non-Western diets, coping and community connection.

### 4.2. Relationship to Current Evidence

The findings align with a broader social determinant of health literature showing that diabetes risk and diabetes care are affected by food environments, economic resources, health-care access, social context and discrimination [14]. Recent young-adult data are particularly relevant: in a cross-sectional analysis of 14,217 adults aged 18–35 years in the All of Us Research Program, 5.5% had prediabetes or T2D, and higher loneliness, stress and discrimination were associated with higher odds of prediabetes/T2D [28]. This supports the importance of taking the participant’s isolation and mental health strain seriously as diabetes prevention issues rather than treating them as secondary emotions outside the intervention’s scope.

The participant’s desire for culturally relevant nutrition guidance is also consistent with recent DPP and diabetes education literature. A 2025 qualitative study of Hispanic adults participating in the National DPP emphasized the emotional impact of prediabetes awareness and the value of language and cultural congruence [29]. Evidence from South Asian-focused lifestyle interventions suggests that culturally bespoke programs can support weight and metabolic outcomes, yet effects vary, and cultural tailoring alone does not guarantee equity [21]. This is important for SSBC: adding South Asian recipes would be useful, but insufficient if the program does not also address student food access, family nutrition literacy, peer support and mental health.

The participant’s cost and campus dining barriers are consistent with implementation evidence indicating that cost, healthy food access, time and clear information affect participation and adherence in diabetes prevention and management programs [30]. The case also resonates with Canadian evidence and policy reports on Black and South Asian populations, which identify low income, food insecurity, limited healthcare access and cultural barriers as recurring determinants of diabetes outcomes [31].

### 4.3. Contribution and Novelty

A single case cannot demonstrate prevalence, comparative effectiveness or subgroup effects. Its novelty is more specific and more defensible: it provides a longitudinal, participant-level equity analysis inside an established Canadian DPP, using interviews and journals before, during and after participation to identify how intersecting factors—South Asian food practices, young adulthood, university residence, cost constraints, social isolation and prediabetes-related distress—shaped engagement with a standard intervention. Prior SSBC studies have established program reach, effectiveness, intervention content and equity-oriented program-development steps [22,23,24]. This case adds an in-depth account of what those equity goals look like at the point of lived program use.

### 4.4. Practical Implications for DPP Design

Four practical implications follow. First, DPPs should develop modular cultural nutrition resources rather than a fixed, Western-centric set. These resources should include non-Western staple foods, common preparation methods, family-based label-reading support and realistic substitutions that preserve cultural connection. Second, programs serving young adults should include age-specific modules on irregular routines, campus dining, social eating, low-cost meals and managing cravings in peer settings. Third, peer connection should be treated as a prevention mechanism: optional support groups or moderated communities may reduce isolation and normalize prediabetes management among younger participants. Fourth, coach training should preserve motivational interviewing while adding structured content on cultural safety, stigma, diabetes distress, referral pathways and mental health-responsive coaching. These recommendations are compatible with equity frameworks such as ConNECT, which emphasizes context, navigation, equity, community and trust in behavioral medicine [32].

### 4.5. Strengths and Limitations

The main strength of this study is temporal depth. Collecting data before, during and after the intervention allowed the analysis to examine how barriers persisted, changed or became more visible during program participation. The combination of two interviews, six session-day journals, and two non-session journals provided a richer account than a single exit interview would have. Open-ended questions allowed the participant to describe thoughts, values and emotional responses related to SSBC, prediabetes, diet and exercise.

The limitations are substantial. One participant cannot represent South Asian people, young adults, gender-diverse people, students or equity-deserving groups. Treating this participant as representative would reproduce tokenism rather than challenge it. The findings are therefore analytic and hypothesis-generating. The study also relied on researcher-designed interview and journal prompts so that some topics may have been missed or shaped by the research team’s assumptions. The analysis did not include member checking, quantitative outcome data, HbA1c change, dietary intake measures, physical activity accelerometery or comparison with other SSBC participants. Because this case was retrospectively and purposively selected from participants enrolled through the existing SSBC program process and required English proficiency, the findings may understate barriers faced by people with language, immigration, disability, rural access or more severe socioeconomic constraints. Furthermore, mental health was explored through qualitative self-report rather than a validated psychological assessment; therefore, the findings reflect the participant’s reported emotional experiences and should not be interpreted as evidence of a clinical mental health condition or deterioration.

### 4.6. Future Directions

Future research should test whether the program refinements suggested by this case improve reach, retention, satisfaction, behavior change and glycemic outcomes among equity-deserving participants. The next step should not be another isolated case alone. A stronger design would combine qualitative longitudinal cases with purposive sampling across age, ethnicity, gender identity, socioeconomic position, language, geography and food environment, alongside implementation outcomes such as acceptability, appropriateness, feasibility, fidelity, reach and maintenance. SSBC and similar DPPs should also disaggregate recruitment, retention and outcome data by equity-relevant variables to identify where exclusion occurs and whether adaptations reduce it.

## 5. Conclusions

This equity-oriented qualitative case study identifies how a young adult South Asian, male-identifying participant experienced prediabetes prevention within SSBC. Supportive, non-judgmental coaching was a clear strength and helped create accountability and motivation. However, culturally irrelevant dietary resources, student food constraints, cost, delayed dietetic access, lack of peer support and mental health strain limited the fit of the standard intervention. The key conclusion is that inclusive diabetes prevention requires more than offering the same evidence-based program to everyone. Programs must examine whose lives their assumptions fit, whose barriers remain invisible and what practical adaptations are needed to make prevention usable for participants at elevated social and structural risk.

## Figures and Tables

**Table 1:** Case profile and data sources.

Domain	Details Retained from the Study Record
Study site and program	Small Steps for Big Changes, a diabetes prevention program developed at the University of British Columbia and delivered as the standard SSBC intervention.
Data collection period	July–September 2022, concurrent with the participant’s intervention experience.
Participant profile	Young adult; self-identified as male; South Asian ethnic background.
Eligibility for case study	Enrolled in SSBC; 18–35 years old; self-identified as male and non-White; able to speak, read and write English.
Program eligibility pathway	HbA1c 5.7% to 6.4% confirmed by a medical laboratory; the participant’s HbA1c result was obtained through their family physician.
Intervention exposure	Standard 4-week intensive phase with one-on-one motivational interviewing-based counseling and supervised exercise; fitness tracker and mobile app self-monitoring; planned 12-month follow-up phase.
Qualitative dataset	Two Zoom interviews lasting approximately 45 and 30 min; six session-day Qualtrics journal entries; two optional non-session journal entries.

**Table 2:** Themes, participant evidence and equity-oriented program implications.

Theme	Observed Barrier or Facilitator	Illustrative Participant Language	Program-Level Implication
Lack of access	Culturally irrelevant dietary information and nutrition literacy gaps.	“Very much not catered towards an ethnic diet”; “my family doesn’t know how to read nutritional values.”	Develop culturally adaptable food examples, label-reading supports and family-oriented resources for non-Western dietary patterns.
Student food environment	Campus dining and social events made healthy choices costly, inconvenient or socially isolating.	“Purely white flour, white paste, red meat”; “always free pizza.”	Include student-specific planning, dining-hall navigation and low-cost substitution strategies.
Healthcare access	Approximately one-year wait for dietician; reliance on self-directed searching.	“Figure out stuff by myself”; “pioneer new ways,…, as a student.”	Create interim, evidence-based resource pathways and referral templates for participants awaiting dietetic care.
Life-stage and resource-related constraints	Young adult routines and limited funds reduced feasibility.	“Older adults have a lot more routine”; “without paying even more of my own money.”	Design age-responsive modules for unpredictable schedules, low budgets and transition-to-adulthood routines.
Lack of support	No known peers managing prediabetes; isolation.	“Other young people managing prediabetes.”	Offer optional peer groups, community matching or moderated discussion spaces.
Mental health implications	Prediabetes and behavior change triggered guilt, shame, stress and loss of food-related joy.	“Overwhelming guilt”; “finding other sources of joy.”	Integrate coping strategies, stigma-sensitive language and mental health referral information.
SSBC positive takeaways	Coach support, non-judgmental style, accountability and aerobic exercise education were valued.	“Very supportive and non-judgemental”; “accountability factor.”	Preserve motivational interviewing-informed coaching while expanding coach training for cultural responsiveness and mental health-responsive support.

## Data Availability

The qualitative data presented in this study are available on reasonable request from the corresponding author. The data are not publicly available due to privacy and ethical restrictions, as interview and journal materials may contain sensitive or potentially identifiable information.

## Supplementary Materials

The following supporting information can be downloaded at https://www.mdpi.com/article/10.3390/healthcare14203432/s1, Supplementary File S1: Journal Entry Prompts Used During the Small Steps for Big Changes (SSBC) Intervention.

## Abbreviations

The following abbreviations are used in this manuscript:

DPP	Diabetes prevention program
T2D	Type 2 diabetes
SSBC	Small Steps for Big Changes
