# Supplementary material for: Understanding How to Make Type 2 Diabetes Prevention Programs More Inclusive: An Equity-Oriented Qualitative Case Study of Small Steps for Big Changes
Source: Healthcare (Basel). 2026 Oct 14;14(20):3432. doi: 10.3390/healthcare14203432 (PMC13704304; doi:10.3390/healthcare14203432)
Supplement: Supplementary file 1 [file healthcare-14-03432-s001.zip › healthcare-4500836-supplementary.pdf]

Supplementary File S1

Journal Entry Prompts Used During the Small Steps for Big Changes (SSBC) Intervention

The participant completed one structured journal entry following each intervention session using Qualtrics. Additional journal entries could be completed voluntarily on non-session days throughout participation. The prompts below were used to guide participant reflection during the study.

Journal Entry 1 – Introduction

1. Describe how your first session went.

2. Describe your first interaction with your coach.

3. If you and your coach talked about exercise during this session, please journal about how these conversations felt. For example, did the exercises apply to, or were relevant to, your daily activities?

4. Describe how this session was or was not culturally relevant to you.

5. Describe how this session was or was not age-relevant to you.

6. Describe how this session was or was not relevant to your gender identity.

7. In this session, was there anything that you believe was missing?

8. Feel free to use this space for any additional thoughts, comments, or concerns.

Journal Entry 2 – Sugar

1. Describe how your second session went.

2. How did the session allow you to reflect on sugar intake in your own life?

3. Describe how this session was or was not culturally relevant to you.

4. Describe how this session was or was not age-relevant to you.

5. Describe how this session was or was not relevant to your gender identity.

6. In this session, was there anything that you believe was missing?

7. Feel free to use this space for any additional thoughts, comments, or concerns.

Journal Entry 3 – Exercise

1. Describe how your third session went.

2. How might the exercise discussion from this session be implemented into your daily life or routine?

3. Describe how this session was or was not culturally relevant to you.

4. Describe how this session was or was not age-relevant to you.

5. Describe how this session was or was not relevant to your gender identity.

6. In this session, was there anything that you believe was missing?

7. Feel free to use this space for any additional thoughts, comments, or concerns.

Journal Entry 4 – Carbohydrates

1. Describe how your fourth session went.

2. Describe how the information on carbohydrates from this session may or may not change your food choices.

3. Describe how this session was or was not culturally relevant to you.

4. Describe how this session was or was not age-relevant to you.

5. Describe how this session was or was not relevant to your gender identity.

6. In this session, was there anything that you believe was missing?

7. Feel free to use this space for any additional thoughts, comments, or concerns.

Journal Entry 5 – Physical Activity

1. Describe how your fifth session went.

2. Are there any exercise or activity suggestions that you would have liked to hear in the session?

3. Describe how this session was or was not culturally relevant to you.

4. Describe how this session was or was not age-relevant to you.

5. Describe how this session was or was not relevant to your gender identity.

6. In this session, was there anything that you believe was missing?

7. Feel free to use this space for any additional thoughts, comments, or concerns.

Journal Entry 6 – Success after SSBC

1. Describe how your sixth session went.

2. How will you take your learnings from the program and implement them into your life?

3. Describe the changes you have already made to your life from SSBC.

4. How will you stay motivated to stick with your new behaviours?

5. Describe how this session was or was not culturally relevant to you.

6. Describe how this session was or was not age-relevant to you.

7. Describe how this session was or was not relevant to your gender identity.

8. In this session, was there anything that you believe was missing?

9. Feel free to use this space for any additional thoughts, comments, or concerns.

Optional Non-Session Journal Entries

1. If applicable, how are you applying your Small Steps learnings in your daily life?

2. How is being in Small Steps changing the way you move throughout your daily life?

3. Describe how Small Steps has made an impact on your daily practices.

4. Describe how your exercise and dieting habits have changed since you started Small Steps.

5. Feel free to use this space for any additional thoughts, comments, or concerns.
